# Network-Aware HEFT Scheduling for Grid

**DOI:** 10.1155/2014/317284

**Published:** 2014-01-22

**Authors:** Muhammad Murtaza Yousaf, Michael Welzl

**Affiliations:** ^1^Punjab University College of Information Technology, University of the Punjab, Allama Iqbal (Old) Campus, Lahore, Pakistan; ^2^Networks and Distributed Systems Group, Department of Informatics, University of Oslo, Norway

## Abstract

We present a network-aware HEFT. The original HEFT does not take care of parallel network flows while designing its schedule for a computational environment where computing nodes are physically at distant locations. In the proposed mechanism, such data transfers are stretched to their realistic completion time. A HEFT schedule with stretched data transfers exhibits the realistic *makespan* of the schedule. It is shown how misleading a schedule can be if the impact of parallel data transfers that share a bottleneck is ignored. A network-aware HEFT can be used to yield a benefit for Grid applications.

## 1. Introduction

The grid is a collaborative execution environment with the contributing resources possibly distributed over large geographical domains. To utilize these resources, the grid offers various services which perform resource management, job creation and submission, resource allocation, storage management, and so forth. Resource management service (RMS) is a vital part of the grid execution environment [1], because it performs resource discovery and ensures that resources available in the Grid can be used by the Grid jobs. A scheduler service communicates to the RMS for available resources and makes the best possible schedule which is then communicated to the enactment engine for actual submission and execution.

With the increase in usage of the Grid resources, the need for better and optimized management is also increasing. Existing systems have addressed various issues related to efficient management of the resources, but effective resource scheduling for the Grid based execution environments still requires a lot of attention. For optimized execution of jobs a precise schedule is required so that jobs are executed using the best (e.g., cheapest) available resources and in the minimum possible time. This leads to the need of an RMS with Grid services providing all the required information about resources, their availability, and cost.

A Grid scheduler can realize this only when it takes all the parameters into account that contribute to the placement and execution of a job on the Grid. The parameters which are considered to mainly contribute to the decision making of a scheduler are computation and storage costs at a specific Grid site. Because most of the algorithms used by schedulers today are designed mainly for geographically colocated parallel and distributed environments, they do not properly address the costs involved in data transfer over the network.

A number of efforts have been done in the area of Grid scheduling research [2], which take into account network costs as a contributing parameter and give it an appropriate weightage. When a scheduler makes a scheduling decision by taking into account the network characteristics, significant gains can be achieved in situations when there is a large amount of data to be transferred to a remote site for the execution of a Grid job.

The next section provides background information that motivates the need for network-aware scheduling in Grid and discusses the execution scenarios where network aware scheduling can play a role.

## 2. Motivation for Network-Aware Grid Scheduling

Capacity of a network channel and its data transfer speed are important factors in the selection of a Grid node for execution of an application on the Grid. Today scientists from numerous scientific domains are making use of resources on the Grid [3]. It is necessary to have schedulers which take into account network capabilities and allocate proper weightage when making a decision of allocation of a compute node to an activity. Network-aware Grid scheduling is important for efficient utilization of computing resources and is quite valuable to all scientific domains utilizing the Grid. A few of these scientific domains are listed below with a brief look into them.

### 2.1. Data Intensive Applications

The Grid is an ideal playground for scientists who execute highly data intensive application for their *in-silico* experiments quite often [3]. One common example of the scientific communities making use of the Grid resources is that of bioinformatics and high energy physics [2]. Scientists in these domains usually execute their compute intensive applications on the Grid, which need high communication speeds and reliability. Due to the heterogeneous and geographically distributed nature of the resources in the Grid it is not possible for the users to have direct access to all the resources. Thus, they have to rely on the decisions taken by the resource manager about the mapping of activities to specific Grid nodes. Here the job of a scheduler is critical and only an optimized schedule produced by the scheduler can guarantee an optimized execution of the experiment.

### 2.2. Replica Management Systems

The Grid has gained fame for its potential to solve large computational problems, which could not have been solved without a huge collection of dedicated resources. The resources available to the Grid users for the execution of jobs in a Grid environment are not collocated and can be dispersed over remote geographical areas. For applications which need large datasets for their processing it is important to have the required data at locations from where it can be accessed easily [4]. The Large Hadron Collider (LHC) is the largest particle accelerator being built at CERN and is supposed to generate petabytes of data in minutes. This leads to the need for replicas to be created for the application on such storage elements which are easily accessible and ensure a certain degree of reliability too. In such scenarios, it is important for Grid schedulers to not only locate a replica for the successful execution but to make a decision of how to use the replica in an efficient manner. This might involve decisions like moving the job to the site containing data, moving the data to the site where the job is running, or even a complete shift of data and application code to a third location [2].

### 2.3. Real-Time Processing Applications

Many highly data intensive applications today require a real-time or near real-time response from the execution system [5]. In such applications the data produced and transported over the data channels needs very fast transmission. A warfare simulation environment is an example of such a scenario where there is a need for real-time response from the system. A correct simulation of the war field is only possible when the data among the processing sites is provided in real-time. Scheduling strategies applied in localized parallel processing environments do not face the problem of low speed data connectivity. Modern network infrastructure has somehow bridged the gap between localized parallel processing environments and that of geographically distributed parallel processing environments. Dedicated high-speed links are, however, rarely available among Grid sites. Thus, the schedulers have a key role in using the existing network infrastructure to maintain the best possible performance for such demanding applications.

## 3. Taxonomy and Classification of Grid Scheduling Techniques

Grid scheduling techniques are classified [2] into various types according to the scheduling mechanism and algorithms adopted by them. These systems have evolved over time and provide different functional characteristics in certain execution environments.

Scheduling algorithms are classified based on the strategy adopted by them for job scheduling and the circumstances in which the scheduler gives best performance. The classification criteria thus take into account the domain in which they are used, this can be a local execution environment (single Grid site) or a global execution environment (multiple Grid sites). A local Grid job scheduling strategy is usually quite simple, while the global job scheduling mechanism can further be classified as static and dynamic. A static Grid scheduling process is a process in which scheduling data about all the jobs and the resources is already available. Static scheduling in the global execution environments is again relatively simple, and the execution schedules provided using this strategy generally perform well. Dynamic Grid scheduling algorithms are more adapted to the changing nature of the Grid and are used to produce execution schedules when there is insufficient information about the resources of the Grid and also about the jobs which are to be scheduled.

## 4. Related Work

The research community involved in Grid scheduling has come up with several solutions which take into account network conditions before they schedule jobs for execution in the Grid. Here, a short survey of the Grid schedulers which take into account the network conditions and capacity when making schedules for job execution is presented.

### 4.1. Network Bandwidth-Aware Grid Resource Brokerage

**Figure 1:**
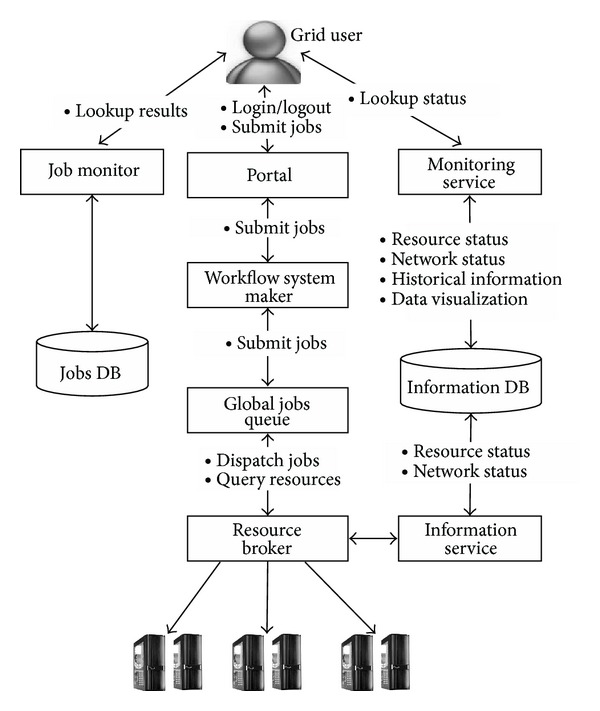
Resource brokerage with network bandwidth-aware Grid scheduling (taken from [6]).

Yang et al. [6] conducted their work on network bandwidth aware resource brokerage by calculating the computing power of a specific grid site by considering the computational power, memory and also the network connectivity available at the site. The architecture of the resource broker based system is shown in Figure 1.

(*1)  Functionality of the Resource Broker*. Users submit their job for execution in the Grid environment. These requests are taken up by the Grid portal, which collects the information about the current status of other resources and also the jobs already running in the Grid environment. Once it finds the resources available for the submitted job it allocates them to the job (resource binding). The scheduler is then given the job for submission to the Grid. Results are collected and propagated back to the resource broker for later usage. Users can also get the results from the portal. As the Grid environment is usually multiplatform, it is hard to get the resource status information from this cross platform environment. The implemented architecture also offers a service that can be used to collect the information for resources, the workload of jobs, and other network conditions related to all the distributed locations in the Grid.

(*2)  Network Bandwidth Aware Scheduler*. The scheduling mechanism uses statistically calculated values based on the compute power *TP* which is a combination of the CPU power, memory power, and the network strength of the site to the outside world. Information about the total computational power *TP* for each of the sites in the Grid is collected by the resource broker. Users who want to use the Grid specify an input value for the compute power they need which is compared to the available resources in the Grid, and job allocation is done to a Grid site which has enough computational resources.

Based on the various parameters chosen, a specific formula is used which provides the total average computing power *ATP* for each Grid site (also called the *ranking* of the site). Afterwards, using that value for each site, the Grid scheduling algorithm is run to perform allocations.

The parameter used in the calculation of *ATP* for the network effect is called performance effect ratio for network (*a*_*NE*_). Only two (*a*_*NE*_) ratios are used in the formula, one is for gigabit and another is for a different setting which is not very realistic. During the experiments it is assumed that all Grid sites are fully connected with direct network links between them. The same bandwidth is assumed for both ways between any two sites; this means that the same data rate is assumed in forward and backward directions, which can be quite unrealistic. This system also does not incorporate the fact that there can be two or more parallel data transfers on the same path.

### 4.2. Data Intensive and Network Aware DIANA Scheduling

The DIANA [2] scheduler takes into account the data processing power and the network characteristics of the Grid environment when making a scheduling decision. The proposed system is based on a study of existing resource management systems and was strongly influenced by the scheduling and resource management systems presented in [1, 7]. The architecture emphasizes that it is not important to send either data or the application code to only the sites where one of them already resides. Data and application code both can be transferred to a third location which is cheaper in terms of data transfer, and the site is sufficiently fast and capacitive to contain and execute the desired application.

**Figure 2:**
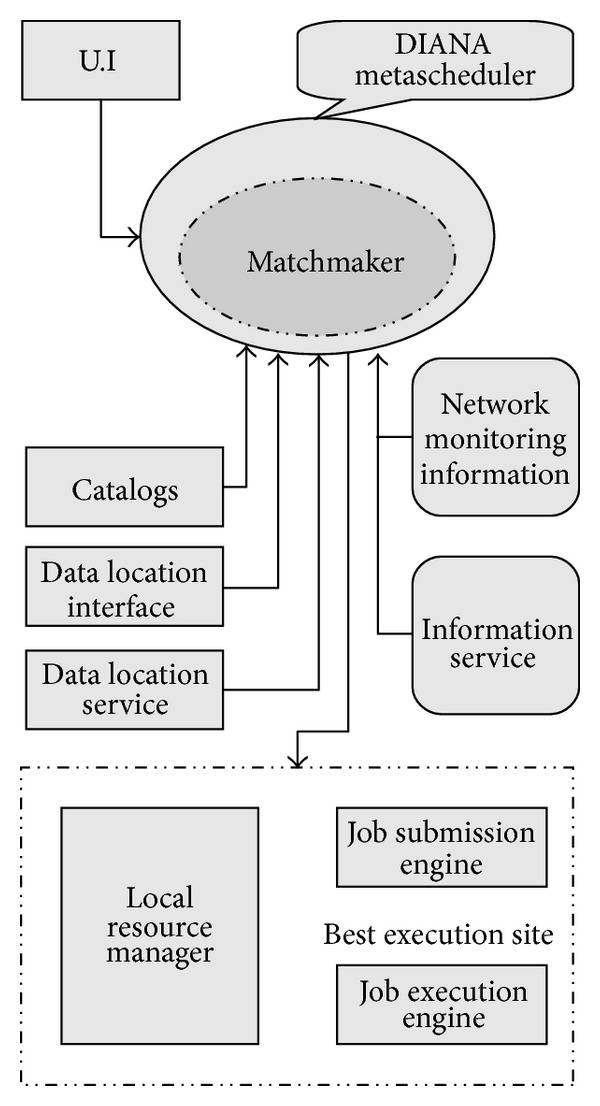
DIANA scheduler (taken from [2]).

The proposed scheduling system, depicted in Figure 2, requires the following parametric information to reach a decision about the schedule it has to make.

- Bandwidth latencies, packet loss, jitter, and anomalies in the network.
- Available computing cycles.
- Site loads and respective job queues.
- Size of the application executable and processing data.

Main objectives set forth are to optimize the queuing time and site load and to improve the transfer time for data executables and the results obtained thereby. A scheduling decision is made on the basis of three costs, which are network cost, computation cost, and data transfer cost (shown in (1)).

(1) Total  Cost=Network  Cost+Computation  Cost +Data  Transfer  Cost.

Network cost is a function of RTT (round trip time), loss, jitter, and bandwidth and is measured as

(2) $$\begin{array}{c} \text{Net}\;\text{Cost}\propto \frac{\text{Losses}}{\text{Bandwidth}}, \end{array}$$

where

(3) $$\begin{array}{c} \text{Losses}=RTT\times W_{1}+\text{Loss}\times W_{2}+\text{Jitter}\times W_{3}. \end{array}$$

A data transfer cost is the cost of transferring input data for the application, output data by the application, and executables. The cost of each type of data is further calculated by multiplying the amount of data by the network cost and the result is further multiplied by some weight as shown in (4):

(4) Data  Transfer  Cost=W4×ID×NC(i−j)+  W5 ×(AD+OD)×NC(local−j)+W6 ×(N−(j)×(ID+AD)+OD)×NC(j),

where ID, AD, OD, and NC represent input data, application data, output data, and network cost, respectively. The weights in both equations are used to increase or decrease the importance of certain factors.

The DIANA scheduler considers most of the characteristics of a system while calculating the total computation cost over it, but does not provide any algorithm or heuristic to schedule all the jobs of a complete workflow based application. This should be done by taking care of all data and control dependencies in order to minimize the overall completion time of the whole application. In DIANA it is just assumed that one job is scheduled where it might need to transfer input data, output data, and the executables and then the best site is found for its allocation on the basis of the above mentioned calculations. Further, the DIANA scheduler also ignores the possibilities of parallel data transfers which could result in having two or more flows passing through same bottleneck, hence affecting each other and ultimately increasing the overall data transfer time.

### 4.3. Conclusion of Related Work

To the best of our knowledge, not much work in the area of network aware scheduling exists except for the approaches discussed in previous sections. As one other example, the authors of [8] proposed an algorithm and architecture for network aware scheduling. In this approach, the network link latency is measured, and then this latency is used to calculate the effective bandwidth of the link. The authors of [9] have presented a multilayer traffic engineering procedure for enabling Grid networks to self-adjust in response to changing scenarios. This proposal is based on monitoring the state of resources and on task migration.

In general, network characteristics are used in a very simplistic and nonrealistic way by state-of-the-art Grid schedulers. But overall, none of the mechanisms consider the shared bottlenecks.

## 5. A Network-Aware HEFT

The proposed mechanism of network-aware Grid scheduling is actually based on the HEFT (heterogeneous-earliest-finish-time) algorithm [10], which is a task scheduling algorithm for heterogeneous computing environments. In the following sections, after explaining the basics of the task scheduling problem, a brief overview of HEFT scheduling algorithm will be presented, followed by some suggested network enhancements, and finally, the complete methodology for Grid scheduling will be described. This methodology takes all the dynamics of real networks into account and provides an efficient and realistic schedule for workflow based Grid applications.

### 5.1. Task Scheduling Problem

**Figure 3:**
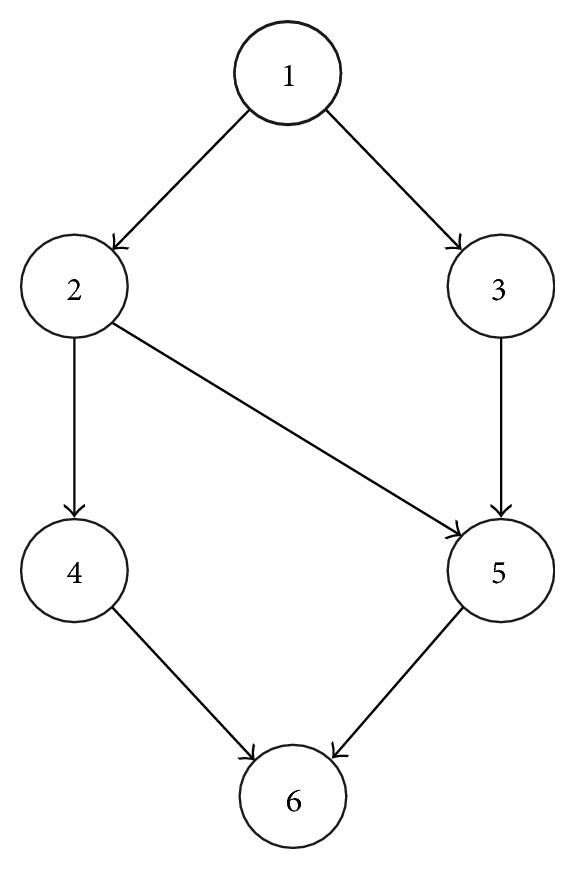
A sample DAG.

In a common task scheduling problem, the tasks of an application are assigned to computing resources according to their order of execution and data dependencies. Generally, such a problem is represented by a DAG (directed acyclic graph), as shown in Figure 3, in such a way that

- nodes represent tasks of the application, and
- edges represent data dependencies between different tasks.

In its simplest form, each node (or task of an application) is labeled with its expected computation time and each edge is labeled with its expected communication time. It is required to provide such a mapping of tasks onto computing resources that minimize the overall completion time without violating the data dependencies among the tasks. This problem of task scheduling is found to be NP-complete [11].

### 5.2. HEFT

The scheduling of different tasks of an application on various resources is a fundamental requirement in a multiprocessing environment. It becomes more important and challenging when resources are heterogeneous in nature and distributed on geographically distant locations, because in such a situation an efficient schedule should also take care of communication infrastructure along with computing resources. There have been many algorithms proposed for task scheduling, but most of them are for homogeneous computing environments. HEFT is generally considered to be one of the most efficient algorithms for heterogeneous computing environments.

For the HEFT algorithm, the task scheduling problem is modeled in the following way [10].

- An application is represented by a DAG (*V*, *E*), where
  - *V* is a set of *v* tasks represented as nodes of DAG and
  - *E* is a set of *e* edges where the source node of each edge must be computed before the execution of its target node.
- The amount of data to be transferred from each task to its dependant task is represented in a Data_(*v*×*v*)_ matrix, where data_(*i*,*j*)_ is the amount of data to be transmitted from task *n*_*i*_ to task *n*_*j*_.
- *Q* is a set of *q* heterogeneous fully connected computing resources.
- *W*_(*v*×*q*)_ is a matrix for computation cost where *w*_(*i*,*j*)_ represents the computation cost of node *n*_*i*_ on computing resource *p*_*j*_.
- *B*_(*q*×*q*)_ is a matrix that holds the data transfer rates between all processors.
- *L* is a set of *q* communication startup costs for each computing resource.

In the above mentioned model, it is assumed that

- all interprocessor communication will be performed without contention.
- computation can be performed with communication.
- task execution of an application is nonpreemptive.

First of all, each task *n*_*i*_ is labeled with its average computation cost $\overset{-}{w_{ı}}$ as calculated by using (5)

(5) $$\begin{array}{c} \overset{-}{w_{ı}}=\overset{q}{\underset{j=1}{\sum }}\frac{w_{i,j}}{q}. \end{array}$$

The communication cost for an edge *e*_*i*,*j*_ from task *n*_*i*_ to task *n*_*j*_, where *n*_*i*_ is scheduled on computing resource *p*_*m*_ and *n*_*j*_ is scheduled on computing resource *p*_*n*_, is calculated with (6), and each edge is labeled with its average communication cost, as calculated by (7):

(6) $$\begin{array}{c} c_{\left(i,j\right)}=L_{m}+\frac{{\text{data}}_{\left(i,j\right)}}{B_{\left(m,n\right)}}, \end{array}$$

(7) $$\begin{array}{c} \overset{-}{c_{\left(ı,ȷ\right)}}={\overset{-}{L}}_{}+\frac{{\text{data}}_{\left(i,j\right)}}{{\overset{-}{B}}_{}}, \end{array}$$

where $\overset{-}{B}$ is the average transfer rate among all computing resources and $\overset{-}{L}$ is the average startup communication time.

After labeling all the nodes and edges, the actual HEFT algorithm is applied. It has two phases.

*Ranking Phase*. During this phase, each node is assigned a rank which determines the scheduling priority of that node. Ranks can be based on an *upward* or *downward* approach. In HEFT, an *upward* rank is assigned for each node. An *upward* rank is recursively calculated upwards for each node starting from *n*_exit_ (the exit node). This is denoted by rank⁡_*u*_ and defined in (8). Further details can be found in [10]. Consider

(8) $$\begin{array}{c} {\mathrm{rank}}_{u}\left(n_{i}\right)=\overset{-}{w_{ı}}+\underset{n_{j}\in \text{succ}(n_{i})}{\max}\left(\overset{-}{c_{\left(ı,ȷ\right)}}+{\mathrm{rank}}_{u}\left(n_{j}\right)\right). \end{array}$$

Here, succ(*n*_*i*_) is a set of immediate successors of node *n*_*i*_. The terminating point of this recursive function is the rank of *n*_exit_, and the rank of *n*_exit_ is equal to its average computation cost as mentioned in (9). Consider

(9) $$\begin{array}{c} {\mathrm{rank}}_{u}\left(n_{\text{exit}}\right)=\overset{-}{w_{\text{exit}}}. \end{array}$$

*Mapping Phase*. Before the start of this phase, all nodes are sorted in the descending order of their *upward* ranks. After that, nodes are picked from the sorted rank list and assigned to computing resources in a way that yields the earliest expected finish time.

### 5.3. Network-Aware HEFT for Grid Scheduling

In this section, the enhancements carried out in order to make HEFT a network aware scheduling algorithm are described. First, a simple model is proposed to estimate the communication cost for each data transfer, and then a methodology is presented which takes care of parallel flows that share the same bottleneck. In the original HEFT it is assumed that, as described in Section 5.2, all interprocessor communication is performed without contention. For real networks, this assumption may not be true. So, a methodology is provided for Grid scheduling that is based on enhanced HEFT (in terms of edge costs) which will provide a realistic schedule by incorporating the characteristics of real networks without changing the foundational behaviors of HEFT.

*Enhancements in HEFT Model*. Generally, in the Grid community it is assumed that a better throughput can be achieved by using multiple TCP sockets at the same time for bulk data transfers in large data intensive applications. Using this approach may provide better performance than using a single TCP connection, but generating too many flows may have an opposite result. It is possible that due to congestion between various different flows, it may increase the total execution time. A simple model of parallel TCP connections has been designed [12] as defined in (10). It models the overall throughput achieved by *N* TCP connections, competing for a fixed capacity *c*. Consider

(10) $$\begin{array}{c} \frac{{\text{throughput}}_{\left(N\right)}}{c}=1-\frac{1}{1+3N}. \end{array}$$

A mechanism to detect shared bottlenecks in the network has been presented in [13]. Here it is proposed to estimate edge costs (data transfer time between different tasks) in a more realistic way by using the model described in (10). In this way, it is suggested to have a matrix *S*_(*q*×*q*)_ of network path capacities in the HEFT model described in Section 5.2, where *s*_*m*,*n*_ represents the path capacity from computing resource *p*_*m*_ to *p*_*n*_.

The communication cost for an edge *e*_*i*,*j*_ from task *n*_*i*_ to task *n*_*j*_, where *n*_*i*_ is scheduled on computing resource *p*_*m*_ and *n*_*j*_ is scheduled on computing resource *p*_*n*_, is calculated with (11), where throughput_(1)_ is the achievable throughput of a single TCP connection using the model presented in (10). Consider

(11) $$\begin{array}{c} c_{\left(i,j\right)}=\frac{{\text{data}}_{\left(i,j\right)}}{{\text{throughput}}_{\left(1\right)}\times s_{\left(m,n\right)}}. \end{array}$$

Before the start of the ranking phase in HEFT, each edge will be labeled by its average communication cost, calculated with (12), where $\overset{-}{S}$ is the average capacity of all paths between the computing resources. Consider

(12) $$\begin{array}{c} c_{\left(i,j\right)}=\frac{{\text{data}}_{\left(i,j\right)}}{{\text{throughput}}_{\left(1\right)}\times \overset{-}{S}}. \end{array}$$

In next section, we provide an example which shows that a schedule produced by the original HEFT algorithm is not realistic and highlight the need for a realistic schedule.

*Realistic Schedule, a Motivating Example*. The HEFT model has a major assumption that interprocessor communication is performed without contention which is not true in realistic scenarios. Parallel flows can affect the performance of each other and cause congestion. This congestion results in packet loss which ultimately increases the data transfer time of a flow over a network path. Here, it is demonstrated with a simple example that a realistic schedule could be much longer than the original schedule designed by HEFT.

**Figure 4:**
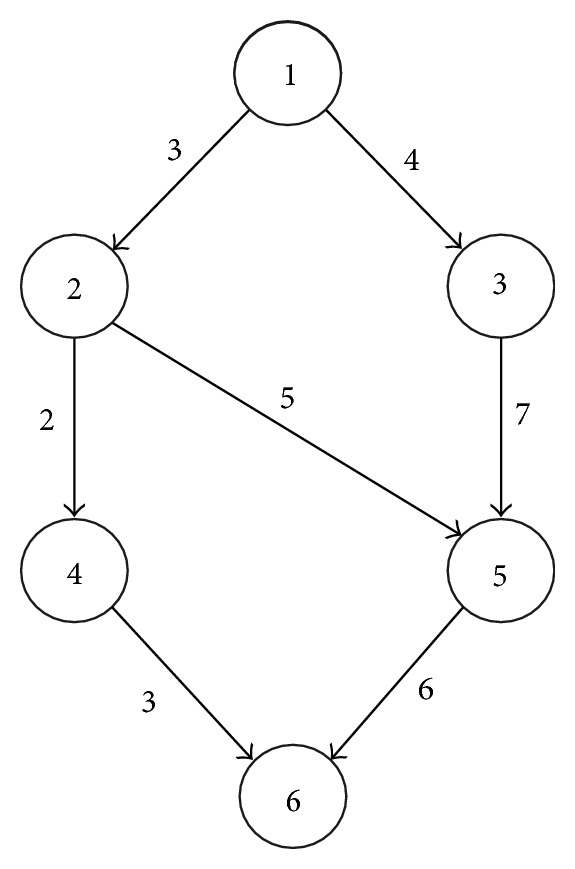
An application DAG with average communication costs.

**Figure 5:**
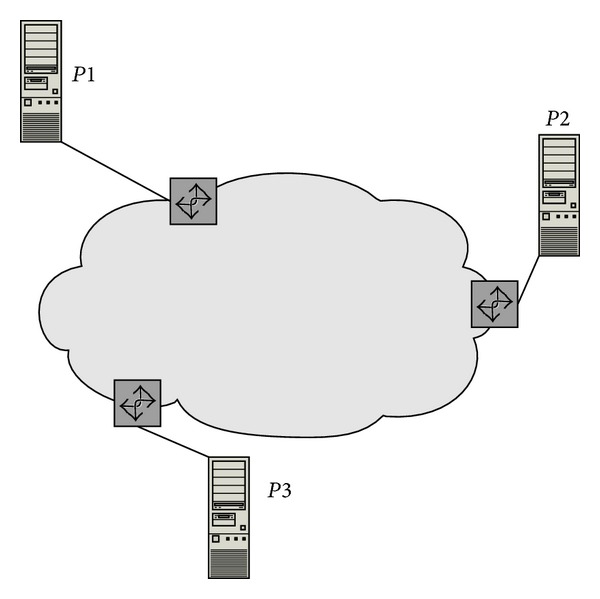
Network topology for DAG example.

Consider the DAG of an application shown in Figure 4 (the example has been taken from the work done for [14]), where each edge is labeled with its average communication cost. Suppose there are three different computing environments available which are connected by a usual wide area network, with the topology depicted in Figure 5. It is further assumed that paths *P*1 − *P*2 and *P*3 − *P*2 share a bottleneck.  Table 1 contains the computation cost of each task on all computing resources.

**Figure 6:**
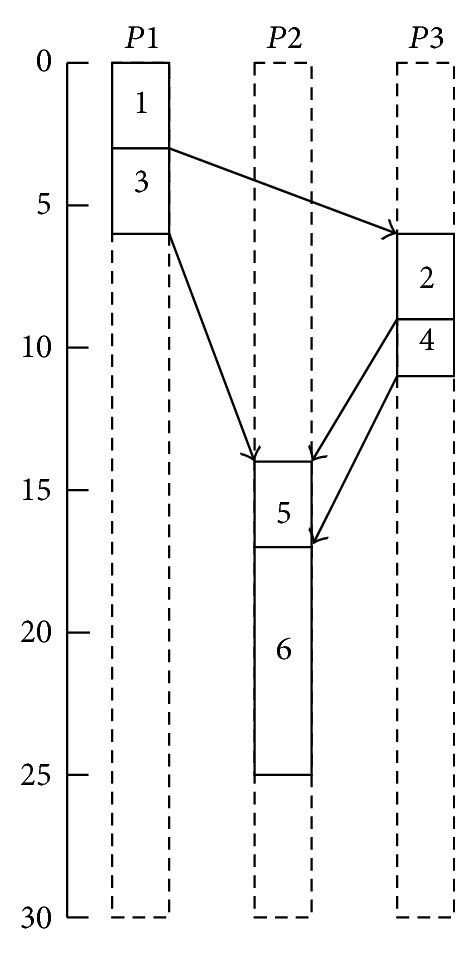
Schedule obtained by HEFT.

After applying HEFT, the schedule presented in Figure 6 is obtained. In this schedule the following four data transfers can be observed:

1. *e*_(1,2)_ from *P*1 to *P*3, starting at second 3 with expected data transfer time of 3 seconds,
2. *e*_(3,5)_ from *P*1 to *P*2, starting at second 6 with expected data transfer time of 7 seconds,
3. *e*_(2,5)_ from *P*3 to *P*2, starting at second 9 with expected data transfer time of 5 seconds,
4. *e*_(4,6)_ from *P*3 to *P*2, starting at second 11 with expected data transfer time of 3 seconds.

The expected data transfer time is picked directly from the application DAG where these costs were estimated with the assumption that there will not be any contention with any other flow. From the schedule presented in Figure 5 and keeping in view the topology depicted in Figure 6 it can be observed that the data transfers *e*_(2,5)_ and *e*_(4,6)_ will be sharing the same network path and they will also share the bottleneck with *e*_(3,5)_. This sharing will ultimately increase the data transfer times of these flows. Hence, the expected data transfer times mentioned in the schedule produced by HEFT are not realistic, and so the *makespan *(overall completion time of the schedule) of 25 seconds cannot be realistic.

**Figure 7:**
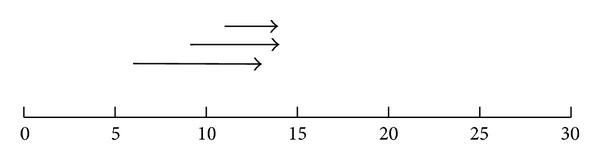
Data transfers with their start times and sharing period in the HEFT schedule.

In order to determine the realistic *makespan* of this schedule, the data transfer times of those flows that share the same network bottleneck will have to be estimated again. This is called *stretching* of flows to their realistic lengths and will be discussed in detail in the following section. Here, the *stretching* of *e*_(2,5)_, *e*_(4,6)_, and *e*_(3,5)_ is demonstrated in a simplistic way. These flows are shown in Figure 7 on a time domain with their start times and the time periods when they will be sharing the bottlenecks in the current schedule.

First, simple theoretical calculations for *stretching* are provided, without assuming the realities of TCP, in the following steps.

- Suppose that the bottleneck capacity is 100 MB/sec and flows *e*_(2,5)_, *e*_(4,6)_, and *e*_(3,5)_ have to transfer 500 MB, 300 MB, and 700 MB, respectively. For the sake of simplicity it is also assumed that 1 MB = 1000^2^ bytes; that is, we ignore the fact that 1 MB is normally 1024^2^ bytes.
- Flow *e*_(3,5)_ will be alone from the 6th second till the 6th second, and it will be able to transfer 300 MB in these 3 seconds. The data left by flow *e*_(3,5)_ at the 9th second will be 400 MB.
- From the 9th second, flows *e*_(3,5)_ and *e*_(2,5)_ will be sharing the same bottleneck for the next 2 seconds until the 11th second, and each flow will only get one half of the whole capacity. This means that each flow will be able to transfer 50 MB/sec and both flows will therefore be able to transfer 100 MB each during these 2 seconds. This means that data left by flows *e*_(3,5)_ and *e*_(2,5)_ after the 11th second will be 300 MB and 400 MB, respectively.
- From the 11th second, flows *e*_(3,5)_, *e*_(2,5)_, and *e*_(4,6)_ will be sharing the same bottleneck, and each flow will get only one third of the whole capacity. This means that each flow will be able to transfer (100/3) MB/sec, and therefore each flow will be able to transfer 100 MB every 3 seconds.
- In this way, flows *e*_(3,5)_ and *e*_(4,6)_ will be able to complete their data transfer in the next 9 seconds because each of them needs to transfer 300 MB at this stage; hence the finish time for these flows will be 20.
- At the 20th second only flow *e*_(2,5)_ will be left with 100 MB; hence it will complete its transfer in the next second, and so its finish time will be 21.

**Figure 8:**
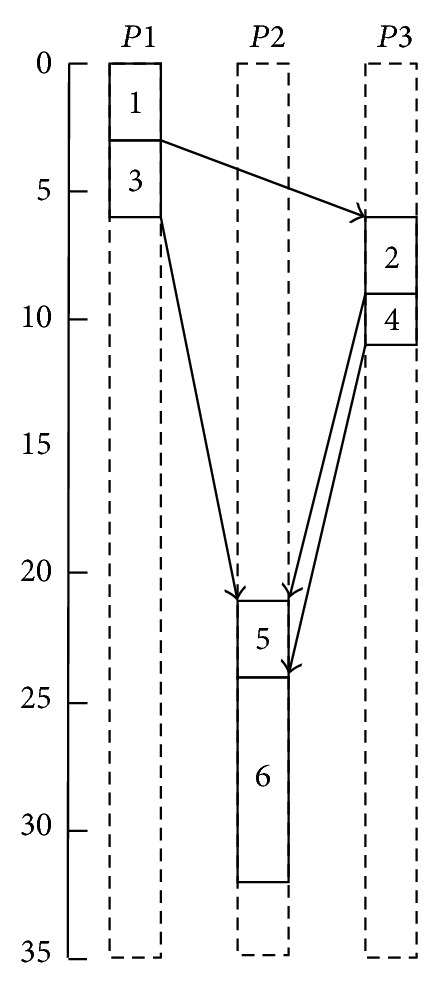
Realistic view of schedule by HEFT.

On the basis of these estimations, a realistic schedule can be visualized in Figure 8 which shows that *makespan* is now 32 (it was 25 in the original schedule provided by HEFT).

In the next step, the throughput model presented in the equation for multiple TCP flows competing for the same bottleneck capacity is used. The calculations were performed just like the theoretical calculations discussed above, and the resulting realistic schedule has a *makespan* of 34.38 which shows an increment of 37.52% in the *makespan* of the original schedule produced by HEFT.

**Figure 9:**
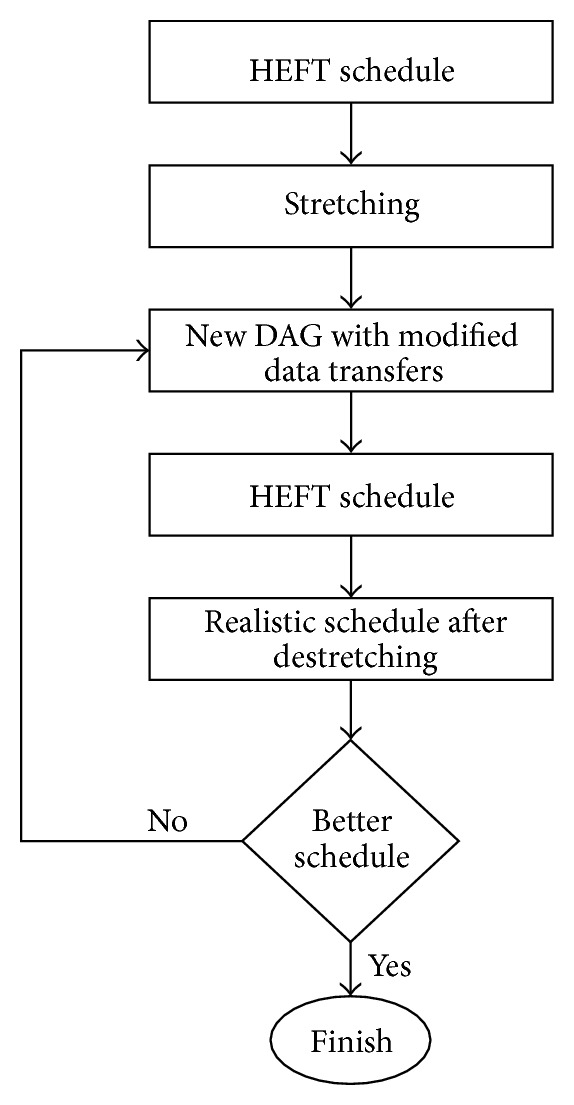
A flow chart of the network-aware HEFT methodology.

*Methodology. *In this section, a methodology is presented to provide a network aware schedule with a realistic *makespan* considering all data transfers over the shared bottlenecks. A flow chart of this methodology is depicted in Figure 9 which consists of five steps. These steps are as follows.

1. In the first step a regular schedule is obtained by applying HEFT on DAG application.
2. In this step, all those data transfers are picked from the schedule that share a bottleneck and these flows are stretched to their realistic lengths in the following manner.
  - A pool of all those flows that shared the same bottleneck is formed, and all these flows are sorted in the ascending order of their start times.
  - A set of independent flows (that do not need to wait for the completion of some other flows) is picked from the pool.
  - The amount of transferable data in the shared period is calculated depending on the number of flows in the shared period. The remaining data to be transferred by each flow in the shared period is calculated for the next shared period.
  - If some flow is expected to be completed within the current shared period then its dependent flows are added after its completion time in the current set of shared flows from the pool.
  - The above two steps are repeated until all flows reach their completion time, and the realistic data transfer time for each flow is found.
3. In this step, all realistic communication costs estimated in step 2 are used in the original DAG instead of the average communication costs estimated in step 1, and a modified DAG is obtained.
4. After getting a DAG with some modified communication costs, standard HEFT is applied again. The main intuition behind this step is that HEFT will try to improve the schedule produced in step 1 so that the new schedule is expected to be better than the original schedule. During the ranking and mapping phase of HEFT, the fact that data transfers might share bottlenecks will be implicitly considered because of modified communication costs. If at this step, HEFT decides on the same mapping of tasks to computing resources as in step 1, then the same realistic schedule that was obtained at the end of step 2. will be produced Alternately, HEFT will produce a new and better mapping of tasks with some of the modified communication costs. These modified costs may interfere with some other communication costs and in this case a new schedule might not be the desired one in terms of its *makespan*. It is also possible that in the new mapping, data transfers with modified costs may not compete with any other data transfer, thus resulting in a better and desired schedule.
5. The schedule produced after step 4 might have some data transfer times that differ from what was calculated in step 2 because of the new way in which data transfers affect each other. For instance, in the new schedule produced after step 4, it is possible that none of the flows that were stretched during step 2 share a bottleneck. Hence, in this step, all data transfers will be realistically estimated and a final schedule is obtained. If the current schedule is better than the original one then the process will terminate, otherwise, control will shift back to step 3 and all steps will be performed again. As already mentioned, task scheduling is an NP-complete problem so there does not exist any unique solution. Hence, we can iterate longer to try to find the best possible schedule. More details will be given in Section 6.2.

## 6. Performance Results and Discussion

Performance results of the network aware scheduling strategy presented in the previous section are presented in this section. Randomly generated application DAGs have been used for performance evaluation. The metric used for performance evaluation is *makespan*, and it is defined as

(13) $$\begin{array}{c} makespan=\text{FT}\left(n_{\text{exit}}\right). \end{array}$$

where FT(*n*_exit_) is the scheduled finish time of the exit node in the DAG.

### 6.1. Random DAGs and Execution Platform

The repository of 1,296 DAGs available at [15] have been used for the performance evaluation, where DAGs were randomy generated according to the following parameters [15, 16].

 
*n*: the number of application tasks (*nodes*) in the DAG.
 Fat: the width of the DAG, that is, the maximum number of tasks in the application that can be performed in parallel. If the value of this parameter is large then the generated DAG will have a high degree of concurrent tasks and if its values are small then there will be a small number of concurrent tasks and the shape of the DAG will be like a chain.
 Density: this parameter deals with the distribution of tasks at different levels of the DAG.
 Jump: this parameter defines the length of different execution paths.

The repository of DAGs was used for their random structures. Communication and computation costs were assigned randomly for all DAGs during all experiments.

For all the experiments, the target computing system used is depicted in Figure 5 with the same assumptions as in Section 5.3.

### 6.2. Simple Example

**Figure 10:**
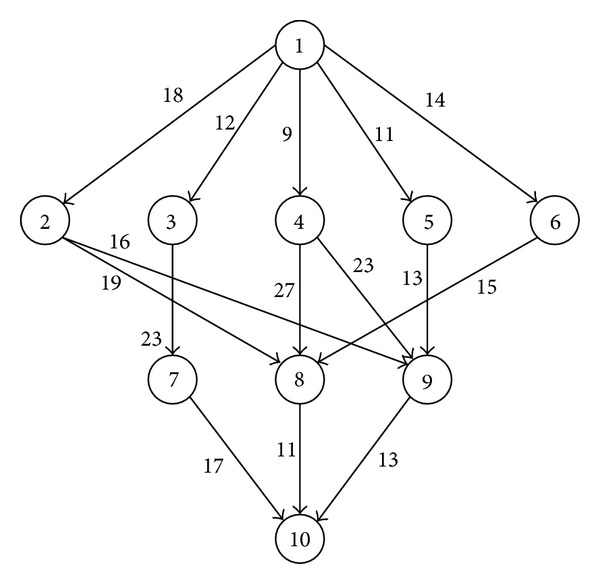
A DAG used for multiple iterations to find minimum *makespan*.

In the first experiment, a sample task graph from [10] was used to analyze the results at each iteration of the network aware scheduling strategy presented in Figure 9. The DAG is depicted in Figure 10. The computation and communication costs were also taken from the original example of [10], and they are shown in Table 2.

**Figure 11:**
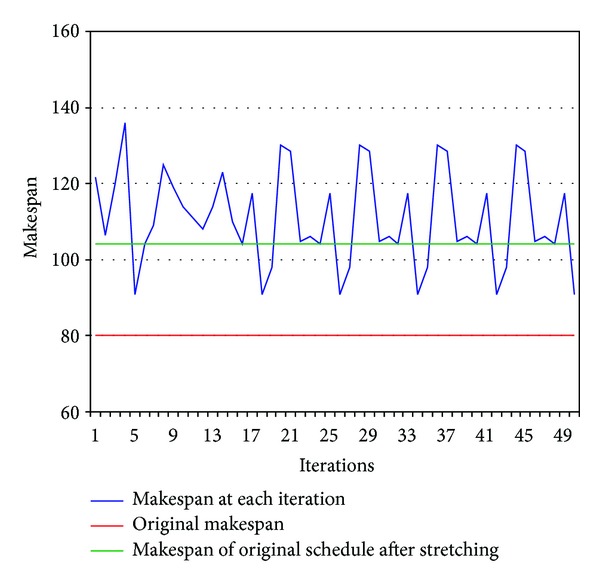
A DAG used for multiple iterations to find the minimum *makespan*.

The *makespan* of the original schedule produced by HEFT is 80. After applying the *stretching* mechanism to the data transfers that share bottlenecks with each other, the *makespan* of the realistic schedule increased from 80 to 104, which is an increase of 30%. The mechanism of network aware scheduling was then repeated 50 times in order to find a better schedule and the resulting *makespan* after each iteration is presented in Figure 11. A better *realistic* schedule (incorporating all shared data transfers and considering their actual data transfer time) was obtained after the 5th iteration of the mechanism. The *makespan* of this schedule was 91, which means a reduction of 12.5% from the original schedule produced by HEFT. It can also be observed from Figure 11 that a pattern of 8 results from the 16th iteration started repeating itself. This behavior was also observed in all other experiments. One possible reason for this repetition could be that, in network aware scheduling, HEFT tries to find the best possible mapping of tasks on the computational resources by considering one particular set of shared data transfers. At each iteration of the mechanism, HEFT tries a new set of shared data transfers. It starts rechecking these sets after checking all combinations with the best mapping of the tasks on the computational resources.

### 6.3. Overall Results

For a detailed performance analysis, each DAG structure in the repository of 1,296 DAGs was used 20 times with randomly generated computation and communication costs, which means that an overall 25,920 different DAGs were used for the experiments. For each randomly generated application graph, 50 iterations of the network aware scheduling strategy were applied. The average results of all DAGs are presented in Table 3. It was observed that the best schedule was obtained on average after the 7th iteration of the network aware scheduling strategy, but this depends on the nature of the application graph. The optimal number of iterations may vary with the structure of the DAG, as will be discussed later. These results showed that on average the *makespan* of original schedule produced by HEFT was increased by 29.65% when data transfers were *stretched* to their realistic lengths, which shows how misleading a schedule could be if the impact of parallel data transfers that share a bottleneck is ignored. After applying the network aware scheduling strategy it was possible to obtain a better schedule. The better schedule reduced the *makespan* by 18.31% on average from the original schedule produced by HEFT.

### 6.4. DAG Size

**Figure 12:**
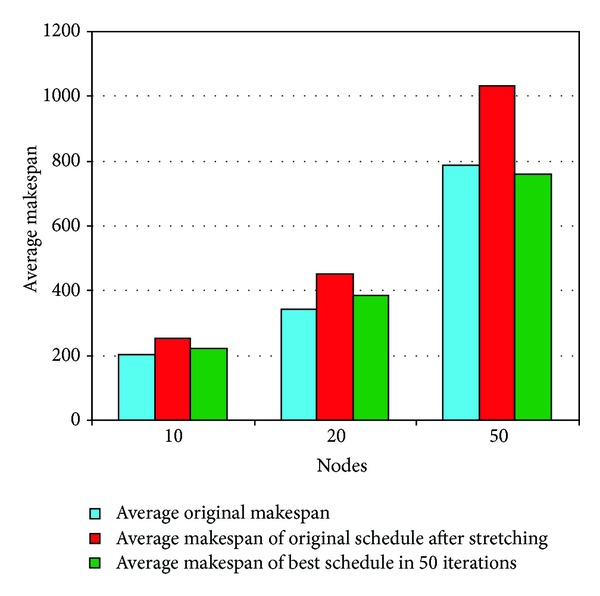
Results for DAGs with different number of nodes.

Figure 12 presents the results with respect to the number of nodes in the application graphs. Three different parameters (10, 20, and 50) were used for the size of DAGs in terms of the number of tasks. It can be observed from Table 4 that the performance gain of the network aware scheduling strategy is proportional to the number of nodes in the DAG. The performance gain in the *makespan* is from 12.67% to 26.43% when the number of nodes in the DAG is increased from 10 to 50. This is significant and very encouraging for large applications, which is usually the case in the Grid scenario.

It can also be observed from Table 4 that the number of iterations required to find a better schedule is proportional to the size of the application graph, and hence for a larger application graph more iterations must be performed to obtain a better schedule with the network aware scheduling strategy. The *makespan* of the original schedule is always significantly misleading, because in all cases it was increased by more than 23% after *stretching* those data transfers that share bottleneck to their realistic lengths.

### 6.5. DAG Structure

In this section, results for different parameters with respect to the structure of the DAGs are discussed. As already stated, four different parameters were used for the variations in the DAG structure during random generation of DAGs. These parameters were varied for DAGs of all sizes (i.e., DAGs with 10, 20, and 50 nodes).

**Figure 13:**
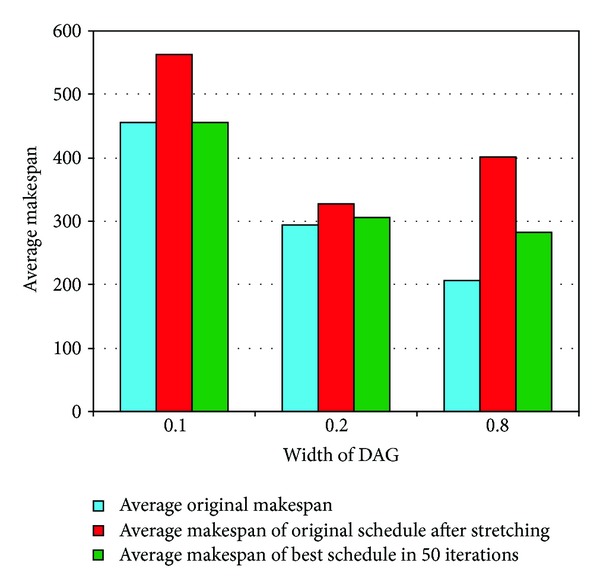
Results for DAGs with different widths.

*Width of the DAG.* The *fat* parameter was used to define the width of the DAG. Three different values (0.1, 0.2, and 0.8) were used for this parameter for all DAGs. A small value of this parameter was used to generate thin graphs which are just like chains, whereas a larger value helped in the generation of compact graphs. The results for DAGs with different values of *fat* are depicted in Figure 13 and the performance gain is presented in Table 5.

The results are quite interesting, as they match the ideology of our network aware scheduling strategy. The DAGs with a large value of the *fat* parameter would be compact and would have a lot of tasks running in parallel, so it can be imagined that in such situations there will be many data transfers going in parallel. If, in such situations, there are many data transfers that share a bottleneck, the realistic *makespan* of the original schedule should be much larger and this is evident in Table 5. It can be observed that the *stretched makespan* is almost twice the original *makespan* provided by the HEFT in the case of compact DAGs. Hence, it can be concluded that it is a big mistake to ignore the stretching factor in DAGs with many parallel tasks.

**Figure 14:**
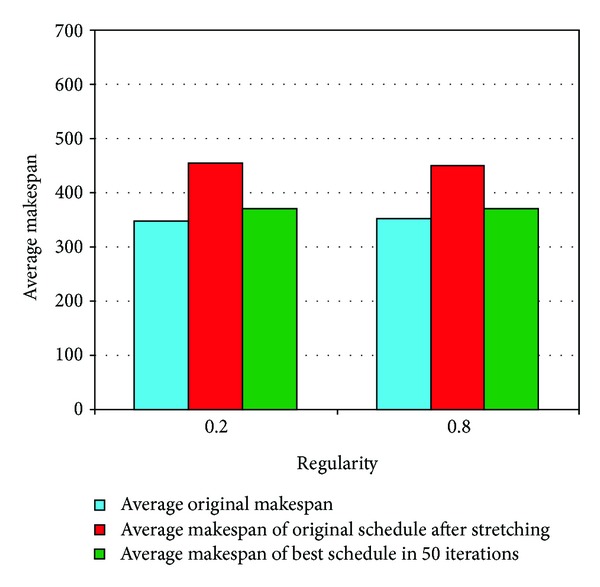
Results for DAGs with different values of *regularity*.

*Regularity.* This parameter was used to define the regularity in the distribution of tasks at different levels of application graphs. Two different values (0.2 and 0.8) were used for this parameter. A small value would mean a less regular distribution whereas a larger value would result in a more regular distribution of tasks at different levels of DAG. The results for DAGs with different values of *regularity* are depicted in Figure 14, and the performance gain is presented in Table 6. It can be observed that the results are almost identical for both values of the *regularity* parameter, and so it can be concluded that the number of tasks at each level of the DAG has more impact (as discussed in the previous section) on the results, and the regularity of the distribution of tasks at each level of the DAG does not matter.

**Figure 15:**
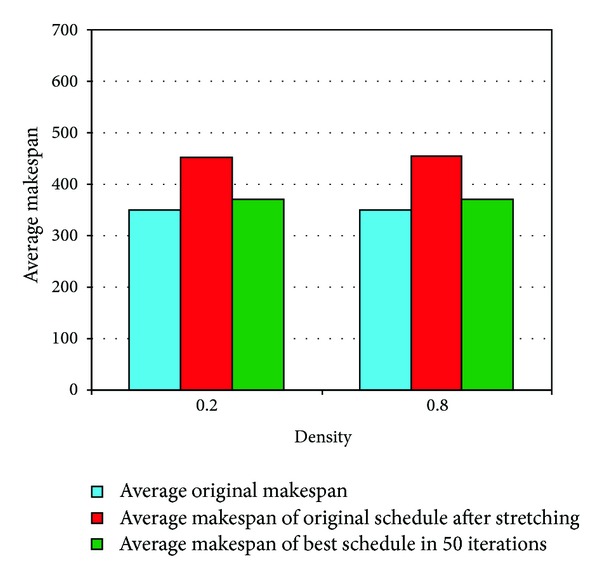
Results for DAGs with different values of *density*.

*Density.* This parameter was used to define the *density* of the DAG. This deals with the number of dependencies between two levels of the DAG. Two different values (0.2 and 0.8) were used for this parameter. The small value of *density* would result in a small number of data transfers, whereas larger values would help in producing a DAG with a lot of communication between different tasks. The results for DAGs with different values of *density* are depicted in Figure 15, and the performance gain is presented in Table 7. It can be observed that the results are almost identical for both values of the *density* parameter.

**Figure 16:**
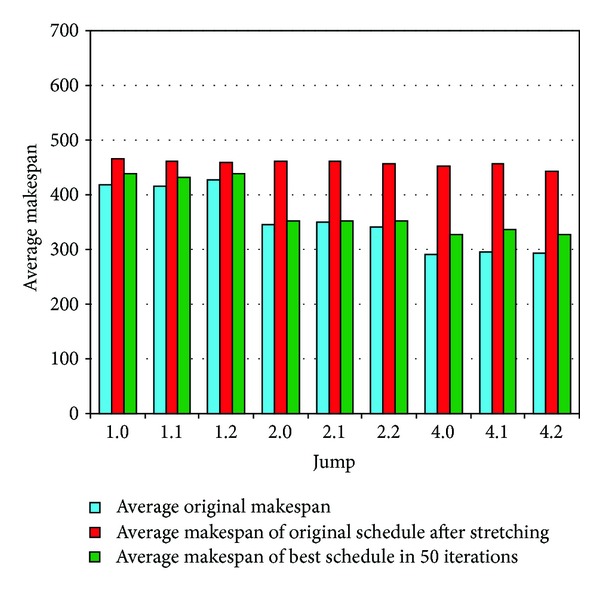
Performance for DAGs with different *jump* values.

*Jump*. This parameter has an impact on the length of execution paths. Nine different values (1.1, 1.2, 1.4, 2.1, 2.2, 2.4, 4.1, 4.2, and 4.4) were used for the *jump* parameter to generate random DAGs. The results for DAGs with different values of *jump* are depicted in Figure 16, and the performance gain is presented in Table 8. The results show a more pronounced performance gain for larger values of *jump* at the cost of requiring a larger number of iterations to find a better schedule. These results are logical because in a longer execution path, data transfers will share a bottleneck for a longer period of time, therefore, resulting in a higher value of realistic *makespan*.

## 7. Conclusion

In this paper, a HEFT based network-aware Grid scheduling mechanism is presented. The first part of this mechanism is an enhancement of the original HEFT model. In this enhancement, an extra matrix of network path capacities is added to HEFT. This matrix is used along with a simple TCP throughput model for “N” parallel TCP connections to calculate the communication costs for all data transfers.

The second part of the network-aware Grid scheduling strategy is to find an optimal realistic schedule that incorporates the parallel data flows that share the same network bottleneck. During this part, first of all, a schedule is obtained using the HEFT algorithm. In this obtained schedule, those data transfers are identified that share the same bottleneck. The original HEFT does not take care of these parallel flows, but in the proposed mechanism, such data transfers are *stretched* to their realistic completion time. A HEFT schedule with *stretched* data transfers exhibits the realistic *makespan* of the schedule. After this step, HEFT is applied again with stretched communication costs and this process is repeated until a better schedule is found. During the evaluation of this strategy it has been found that on average a better schedule was obtained after the 7th iteration of this mechanism. Among other notable results, it was observed that the *makespan* of original schedule produced by HEFT was increased by 29.65% on average when data transfers were *stretched* to their realistic lengths. This shows how misleading a schedule can be if the impact of parallel data transfers that share a bottleneck is ignored. After applying the network aware scheduling strategy it was possible to obtain a better schedule. The better schedule reduced the *makespan* by 18.31% on average in comparison with the original schedule produced by HEFT.

Results also showed that the network-aware Grid scheduling strategy proved to be more effective in case of large DAGs. The performance gain in the *makespan* was from 12.67% to 26.43% when the number of nodes in the DAG was increased from 10 to 50, although the number of iterations to find a better schedule was also increased with the size of the DAG. It was also observed that the *stretched makespan* was almost twice the original *makespan* provided by HEFT in case of compact DAGs. The impact of the *regularity* of nodes at each level of the DAG and the *density* of data transfers among different levels of the DAG were found to be insignificant.

## Figures and Tables

**Table 1:** Computation costs of tasks over computing resources.

Task	*P*1	*P*2	*P*3
1	3	5	7
2	4	8	3
3	3	4	11
4	5	8	2
5	10	3	5
6	5	8	8

**Table 2:** Computation costs.

Task	*P*1	*P*2	*P*3
1	14	16	9
2	13	19	18
3	11	13	19
4	13	8	17
5	12	13	10
6	13	16	9
7	7	15	11
8	5	11	14
9	18	12	20
10	21	7	16

**Table 3:** Average results of all experiments.

Attributes	Results
Average original *makespan*	349.191711
Average *makespan* of original schedule after *makespan*	452.72049
Average *makespan* of best schedule in 50 iterations	369.807617
Average iteration for better schedule	7.099679

**Table 4:** Performance for DAGs with different number of nodes.

Attributes	10 nodes	20 nodes	50 nodes
(Increase in original after)	23.98%	32.47%	31.19%
(Decrease in of better schedule)	12.67%	14.62%	26.43%
Average iteration for better schedule	4.04	7.05	16.22

**Table 5:** Performance for DAGs with different widths.

Attributes	0.1 fat	0.2 fat	0.8 fat
(Increase in original after)	23.31%	11.68%	94.17%
(Decrease in of better schedule)	19.04%	6.39%	29.82%
Average iteration for better schedule	7.31	3.14	12.15

**Table 6:** Performance for DAGs with different *regularity* values.

Attributes	0.2 *regularity *	0.8 *regularity *
(Increase in original after)	31.04%	28.36%
(Decrease in of better schedule)	18.67%	18.07%
Average iteration for better schedule	7.39	6.82

**Table 7:** Performance for DAGs with different *density* values.

Attributes	0.2 *density *	0.8 *density *
(Increase in original after)	29.70%	29.71%
(Decrease in of better schedule)	18.24%	18.47%
Average iteration for better schedule	7.13	7.10

**Table 8:** Performance for DAGs with different *jump* values.

Attributes	1.1 *jump *	1.2 *jump *	1.4 *jump *
(Increase in original after)	11.24%	10.82%	7.89%
(Decrease in of better schedule)	5.99%	6.37%	4.75%
Average iteration for better schedule	3.39	3.76	2.92

	2.1 *jump *	2.2 *jump *	2.4 *jump *

(Increase in original after)	33.21%	33.01%	33.79%
(Decrease in of better schedule)	23.29%	23.52%	22.90%
Average iteration for better schedule	8.03	7.73	7.61

	4.1 *jump *	4.2 *jump *	4.4 *jump *

(Increase in original after)	55.49%	53.77%	51.54%
(Decrease in of better schedule)	27.35%	26.19%	25.99%
Average iteration for better schedule	10.33	10.17	10.41
